# The Relationship Between Negative Life Events and Internet Addiction Disorder Among Adolescents and College Students in China: A Systematic Review and Meta-Analysis

**DOI:** 10.3389/fpsyt.2022.799128

**Published:** 2022-04-27

**Authors:** Jun Wang, Qing-hong Hao, Yang Tu, Yang Wang, Wei Peng, Hui Li, Tian-min Zhu

**Affiliations:** ^1^School of Rehabilitation and Health Preservation, Chengdu University of Traditional Chinese Medicine, Chengdu, China; ^2^School of Acupuncture and Tuina, Chengdu University of Traditional Chinese Medicine, Chengdu, China; ^3^School of Preclinical Medicine, Chengdu University, Chengdu, China

**Keywords:** internet addiction disorder, relationship, adolescents and college students, systematic review, negative life events

## Abstract

**Objective:**

Internet Addiction Disorder (IAD) has become a social problem. Literature suggests that negative life events can cause numerous problematic behaviors and part of them will result in IAD. However, there is a lack of evidence that elucidates the association between negative life events and IAD. Thereby, we performed a comprehensive analysis to further document the relationship between negative life events and IAD among adolescents and college students in China.

**Methods:**

We searched ten electronic databases for relevant articles. We extracted correlation coefficient (r) values from each study and calculated 95% confidence interval (95% CI) after applying Fisher’s *z*. A random-effect or fixed-effect model was applied to analyze the data. Heterogeneity was examined using I^2^ statistics and the Cochran’s Q statistics.

**Results:**

A total of 31 studies were involved in this meta-analysis. Positive correlation was observed between negative life events and IAD. The subtype interpersonal relationship of negative life events was closely associationed with IAD.

**Conclusion:**

There were significant positive association between negative life events and IAD. The findings can be used to guide IAD interventions.

**Systematic Review Registration:**

[https://www.crd.york.ac.uk/prospero/#recordDetails].

## Introduction

Internet Addiction Disorder (IAD) is a behavioral addiction that is described as“uncontrolled impulsive use of the internet that cause serious impairment to the social, psychological, working, and emotional individual adjustments” (1). It has attracted much attention around the world (2). Previous researches suggested that the prevalence of IAD ranges from 1.5 to 8.2% in the United States and Europe, while in Southeast Asia, it could be as high as 20–30% among young people (3). The current study showed that the prevalence of IAD was 7.7% in Chinese college students (4). Literature have indicated that IAD may create sevearal serious problems in adolescents and college students, such as academic problems, school failure, interpersonal relationship problems and family conflict (5, 6). In addition, it may create sleep disorder, impulsive disorder, anxiety, depression, and other health problems (7–9).

Life events are common psychological and social stressors and are depicted as discrete quantifiable circumstances which may result in severe negative impacts (10, 11). Adolescents and college students are a special group. They are more curious but lack mature cognitive control, self-regulatory, and more likely to suffer from learning pressures, peer relationships, environmental adjustment, and other life events (12–14). The Adolescent Self-rating Life Events Checklist (ASLEC) (15) is a self-reported questionnaire which was widely used in China to measure the severity of life stress experienced during the past year of adolescence. It consists of 27 negative events, with 1–5 points for each item, including six factors: interpersonal relationship, learning pressure, punishment, loss, health adaptation, and others. It was used to evaluate the frequency and intensity of stressful life events in adolescents. Research (16–18) has shown that negative life events is an important element that induce or exacerbate various physiological and psychological disturbances (19), which increase suicide risk, criminal behaviors, and addictive behaviors (20–23).

The influencing factors of IAD is still a topic ongoing and under investigation, and negative life events is one of the most important factors. Fan (24) found that negative life events had a significant positive prediction effect on the IAD of college students, that is, the more negative life events they encountered, the more likely they were to develop IAD. Xiao (18) showed that college students with higher scores on internet addiction have significantly higher scores in each dimension and total score of the life events scale than those with lower scores on internet addiction, but Pan et al. found no significant correlation between the two (25). Moreover, in terms of different subtypes of negative life events, the study by Li et al. (26) found that IAD was significantly associated with interpersonal relationship, learning pressure, punishment and health adaptation. However, the results of different studies were not consistent (27, 28). Furthermore, many studies were also limited by differences in diagnostic tools and relative sample sizes, and the results were still inconsistent. Therefore, we conducted a meta-analysis to further clarify the relationship between life events and IAD among adolescents and college students in China.

## Materials and Methods

This systematic review with a meta-analysis was done according to the Preferred Reporting Items for Systematic Review and Meta-Analyses (PRISMA) (21, 22). The review protocol was registered with PROSPERO (CRD42020177316).

### Search Strategy

We searched six international electronic databases include PubMed, Embase, MEDLINE, the Cochrane Library, PsycINFO, ERIC, and four Chinese electronic databases including China National Knowledge Infrastructure (CNKI), Chinese Biomedical Literature Database (CBM), Technology Periodical Database (VIP), and Wan Fang Database. The search strategy was conducted using both Medical Subject Headings (MeSH) terms and free-text words to increase accuracy. The following search terms were used: (“Internet Addiction Disorder”[Mesh] OR “internet addiction” OR “problematic internet use” OR “pathological internet use” OR “internet gaming addiction” OR “internet gaming disorders” OR “excessive internet use” OR “compulsive internet use” OR “internet dependency” OR “computer addiction” OR “internet use disorder”) and (“Life Change Events”[Mesh] OR “life experiences”). In addition to the electronic database, we performed a manual search of the “gray literature” and related literature listed in the bibliography. This search was updated on Aug 2021. The detailed search strategy for PubMed is provided in Supplementary Table 1.

### Selection Criteria

The observational studies were included if: (1) used a validated scale to assess IAD (have been widely used worldwide or have been proved to be valid through reliability and validity tests); (2) reported the relationship between negative life events and IAD; (3) population aged between 12 and 25 years; (4) observational studies published up to Aug 2021; (5) Pearson or Spearman correlation coefficient (r) was available; (6) published in Chinese or English. We excluded studies that included case reports, meeting abstracts, review papers, commentaries, and those with inadequate information.

### Data Extraction

Two reviewers (JW and QHH) independently extracted data with a predefined data extraction form which include the following information: first author, publication year, country, sample size, age, gender, life events measures, IAD measures. Pearson or Spearman correlation coefficient (*r*) between the life events and IAD and mediating factors. Any differences were settled by consensus or discussion.

Spearman correlation coefficients were used for the meta-analyses which were unaffected by monotonic transformations, such as a logarithmic transformation (29). Original studies were log-transformed before analysis and the published Pearson correlation coefficients were converted into Spearman correlation coefficients (30). Since the standard error (SE) depends on the value of the correlation coefficient, a Fisher transformation was used to convert each correlation coefficient (29).

### Quality and Risk of Bias Assessment

We evaluated the quality of the case–control studies by the Newcastle-Ottawa Scale (NOS). Each study was assessed according to 3 domains (selection, comparability, and outcomes) and 8 items with a full score of nine stars. When a study gets more than 6 stars, it is regarded as high-quality research, and those with 4–6 scores are regarded as moderate quality (31, 32). The Agency for Healthcare Research and Quality (AHRQ) for cross-sectional studies, which contains 11 items. An item was scored “0” if it was answered with “no” or “unclear” and “1” if it was answered with “yes.” Those with scores of 8–11 were identified as high quality, and those with scores of 4–7 as medium quality (33). Disagreements were resolved by consensus or a discussion with a third reviewer (YT).

### Statistical Analysis

The association between life events and IAD were assessed using the Pearson product-moment correlation coefficient (*r*- value). “Fisher’s *z* transformation” were used to convert Spearman’s correlation coefficients into the normal distribution. The formula for the transformation is: *Z* = 0.5[*ln* (1 + *r*)−*ln*(1−*r*)] where ln is the natural logarithm (34). In addition, the included studies were weighted according to the magnitude of the respective standard error (SE). The formula for the transformation is: $S\,E=\sqrt{N-3}$ where N represents the respective sample size (34). Furthermore, the degree of variation was estimated by the standard error (SE) and 95% CIs. The *r* < 0.21 indicated poor correlation; 0.21 ≤ *r* < 0.41 was considered average correlation; 0.41 ≤ *r* < 0.61 suggested moderate correlation; 0.61 ≤ *r* < 0.81 meant significant correlation and >0.81 suggested strong correlation (35). Statistical heterogeneity was examined using the I^2^ statistics and the Cochran’s Q statistics. If the values of I^2^ is ≥50% and the *P*-value for the Q-statistic is < 0.05, the study was substantially heterogeneous, and a random effects model was applied to analyze the data. Otherwise, a fixed-effects model was employed. (36). Publication bias was also assessed by conducting the Egger regression asymmetry test and funnel plots (37). The statistical significance was set at *P* < 0.05.

We conducted the meta-analysis with the STATA Version 15.0 Statistical Software.

## Results

### Selected Studies

**FIGURE 1:**
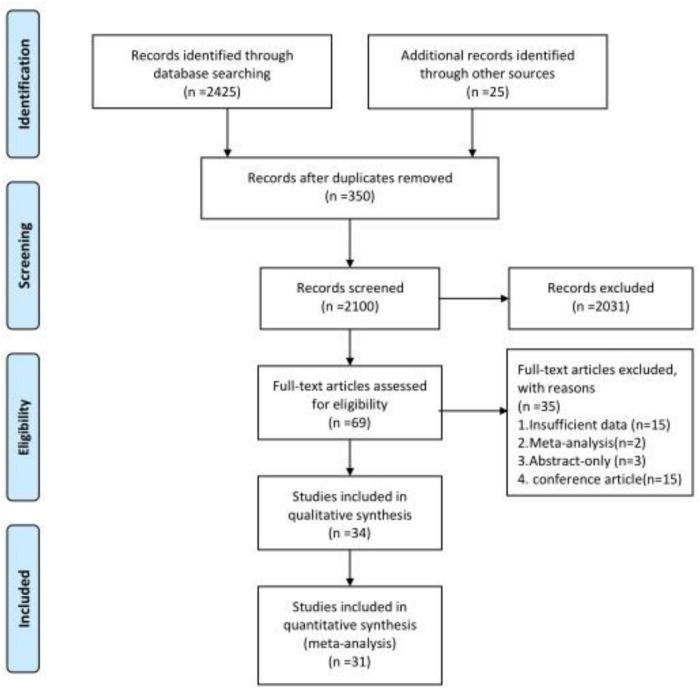
Preferred Reporting Items for Systematic Review and Meta-Analyses (PRISMA) flowchart of included articles.

A total of 2,450 records were identified after database searches, and 2,100 records were left after removing the duplicates. Finally, 69 records were needed for full-text assessments from which 2,031 were excluded after the screening of the titles and abstracts. After reading full-text articles, 31 studies were selected in this systematic review. The study selection process was displayed in Figure 1.

### Study Design Characteristics

Characteristics of the 31 included studies are shown in Table 1. All included studies were observational studies, 30 cross-sectional studies, and 1 case–control study (38–68). The studies included a total of 29,692 participants, ranging from 128 to 10,158 (67, 68). Studies were carried out in China. Chen Internet Addiction Scale (CIAS) were used as the diagnostic tool of IAD in 15 studies (39–42, 44, 46, 50, 52, 54, 57, 58, 60, 62, 64, 68), Revised Chen Internet Addiction Scale (CIAS-R) were used in 5 studies (38, 51, 53, 55, 63), Internet Addiction Impairment Indexes (IAII) were used in 3 studies (43, 65, 66), and 2 studies used the Young’s Diagnostic Questionnaire (YDQ) (49, 61). Two studies used the Chinese version of the Young Internet Addiction Test (IAT) (56, 67), and 1 study used the Internet Addiction Diagnostic Scale (IADS) (45), 3 studies used the Young’s Internet Addiction Scale (YIAS) (47, 48, 59).

**TABLE 1:** Characteristics of the included studies.

References	Sample size	Age range	Study design	Type of assessment (IAD)	Type of assessment (Life events)	Study quality
Yang (44)	200	15–16	Cross-sectional	CIAS	ASLEC	6
Hu (46)	370	18–24	Cross-sectional	CIAS	ASLEC	8
Wang (68)	128	14–22 (18.9 ± 1.6)	Case-control	CIAS	ASLEC	6
Xu (38)	426	N/A	Cross-sectional	CIAS-R	ASLEC	6
Yang (48)	612	18–24(20.58 ± 0.75)	Cross-sectional	CIAS	ASLEC	6
Li (40)	286	12–21	Cross-sectional	CIAS	ASLEC	5
Huang (62)	1043	N/A	Cross-sectional	CIAS	ASLEC	5
Zhou (49)	580	12–19(5.38 ± 0.63)	Cross-sectional	YDQ	ASLEC	6
Dai (56)	324	12–19	Cross-sectional	IAT	ASLEC	6
Cao (43)	611	(13.88 ± 1.52)	Cross-sectional	IAII	ASLEC	6
Hou (63)	602	N/A	Cross-sectional	CIAS-R	ASLEC	6
Li (47)	247	18–23	Cross-sectional	CIAS	ASLEC	6
Xiao (52)	614	N/A	Cross-sectional	CIAS	ASLEC	6
Li (47)	660	12–17(14.4 ± 0.86)	Cross-sectional	YIAS	ASLEC	5
Zhou (66)	787	(21.55 ± 1.36)	Cross-sectional	IAII	ASLEC	6
Cai (65)	479	(17.45 ± 1.00)	Cross-sectional	IAII	ASLEC	6
Chen (53)	317	N/A	Cross-sectional	CIAS-R	ASLEC	5
Wu (57)	826	18–23	Cross-sectional	CIAS	ASLEC	6
Xiao (50)	633	17–25 (20.65 ± 1.316)	Cross-sectional	CIAS	ASLEC	6
Sun (54)	556	12–19	Cross-sectional	CIAS	ASLEC	5
Yan (58)	892	(20.5 ± 1.20)	Cross-sectional	CIAS	ASLEC	6
Hao (64)	441	N/A	Cross-sectional	CIAS	ASLEC	7
Li (55)	395	N/A	Cross-sectional	CIAS-R	ASLEC	7
Yang (59)	3,798	14–17 (15.1 ± 1.5)	Cross-sectional	YIAS	ASLEC	6
Li (61)	483	N/A	Cross-sectional	YDQ	ASLEC	6
Li (48)	998	12–19 (15.15 ± 1.57)	Cross-sectional	YIAS	ASLEC	5
Cao (45)	875	18–22(19.63 ± 0.95)	Cross-sectional	IADS	ASLEC	5
Zhao (67)	10,158	14–24	Cross-sectional	IAT	ASLEC	5
Li (51)	500	N/A	Cross-sectional	CIAS-R	ASLEC	5
Xiao (60)	539	N/A	Cross-sectional	CIAS	ASLEC	6
Guo (41)	312	(21.32 ± 1.53)	Cross-sectional	CIAS	ASLEC	6

*ASLEC, adolescent self-rating life events checklist; CIAS, chen internet addiction scale; CIAS-R, revised chen internet addiction scale; IADS, internet addiction diagnostic scale; IAII, internet addiction impairment indexes; IAT, young internet addiction test; YDQ, the young’s diagnostic questionnaire; YIAS, the young’s internet addiction scale.*

### Quality of the Studies

By using AHRQ and NOS criteria, all included studies scored above four points. Therefore, there were no poor-quality studies in this review (Table 1).

### Main Outcome and Meta-Analysis

We used a random-effects model since the observed heterogeneity was I^2^ > 50% in almost all the outcomes analyzed.

#### Meta-Analysis of the Life Events and Internet Addiction Disorder

**FIGURE 2:**
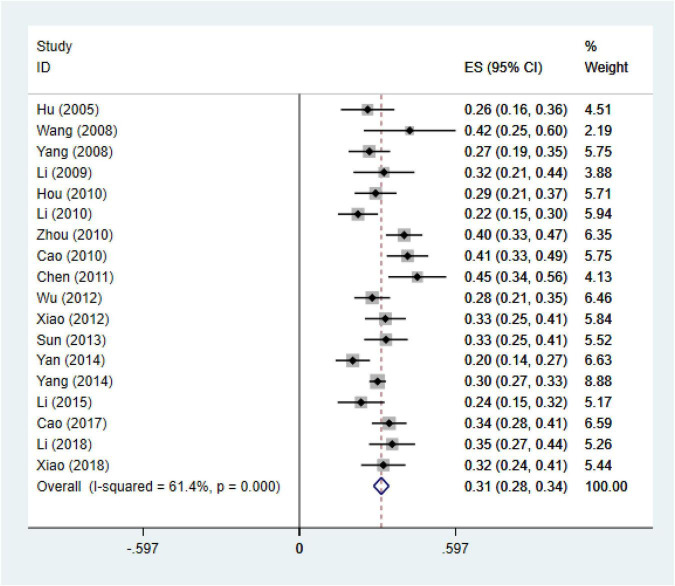
Meta-analysis for the relationship between IAD and negative life events.

**FIGURE 3:**
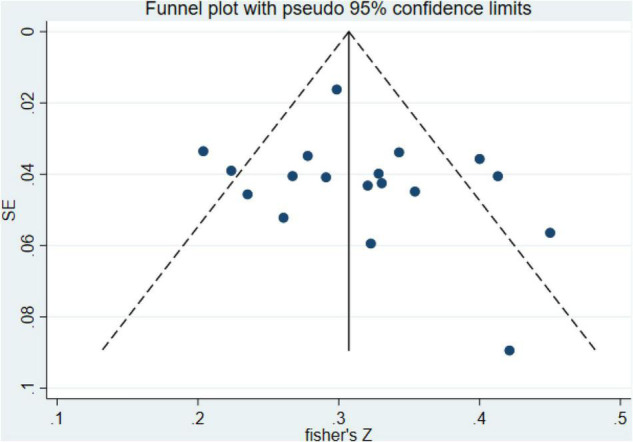
Funnel plot of publication bias for the relationship between IAD and negative life events.

**FIGURE 4:**
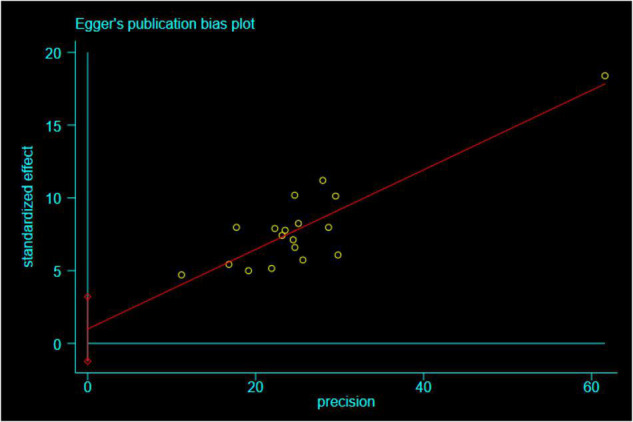
Egger’s publication bias plot for the relationship between IAD and negative life events.

In this meta-analysis, 24 original studies, 17,090 participants were involved to estimate the pooled effect measure between negative life events and IAD (*r* = 0.41; 95% CI 0.31, 0.52), with significant heterogeneity between studies (I^2^ = 97.8%, *p* < 0.01). We conducted sensitivity analyses to identify the source of the heterogeneity, and 6 studies were excluded (38, 41, 48, 51, 55, 64), which only mentioned the diagnostic tools for IAD and didn’t specify clear diagnostic criteria. We recalculated the effect size based on the random effects model after excluding the study (*r* = 0.31; 95% CI 0.28, 0.34) (I^2^ = 61.4%, *p* < 0.01) (Figure 2). It could be seen that although there had been a slight heterogeneity, it was within the acceptable range. In addition, according to the funnel plot chart (Figure 3), studies seem to be distributed symmetrically around its axis, and the results of the Egger statistical tests (*p* = 0.359) indicate that there is no publication bias (Figure 4).

#### Meta-Analysis on Subtypes of Life Events and Internet Addiction Disorder

**FIGURE 5:**
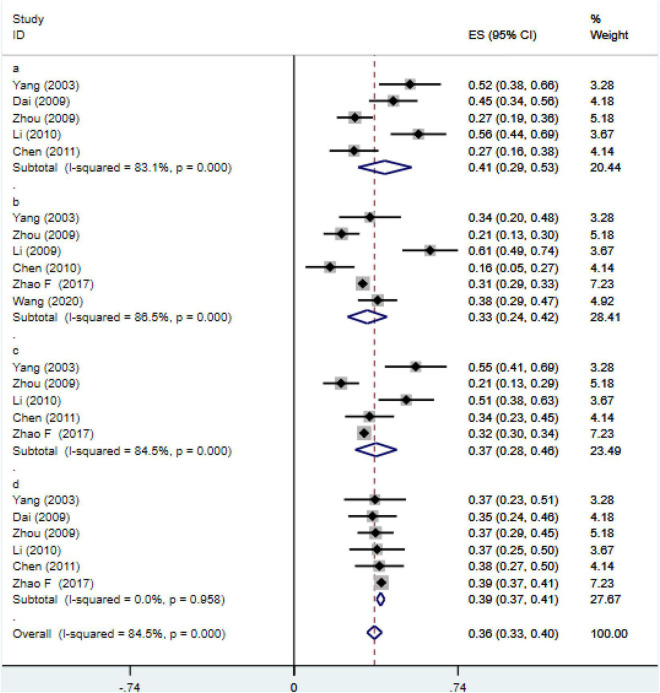
Meta-analysis for the relationship between IAD and subtypes of negative life events. a: interpersonal relationship; b: study pressure; c: punishment; d: others.

A total of 7 studies were included to detect the association between subtypes of life events and IAD (Figure 5). The results showed that there were also obvious heterogeneity in several subtypes (I^2^> 50%, *p* < 0.01). However, after the sensitivity analysis, some studies were removed and meta-analysis was conducted again and found that each study did not substantially change the overall effect sizes. Therefore, random effects were selected to conduct a meta-analysis, and it was concluded that the association between interpersonal relationship and IAD (*r* = 0.41; 95% CI 0.29, 0.53), study pressure (*r* = 0.33, 95% CI 0.24, 0.42), punishment (*r* = 0.37, 95% CI 0.28, 0.46), others (*r* = 0.39, 95% CI 0.37, 0.41).

## Discussion

### Life Events and Internet Addiction Disorder

In recent years, IAD has become an important global mental health problem (69). Negative life events have been proven to have an important role in problematic behaviors, and also have attached further attention (70, 71). Numerous studies have explored the relationship between negative life events and IAD, but the results are still inconsistent (24, 25, 47, 72–74). This review showed that there was a significant positive correlation between negative life events and IAD. Additionally, the subtypes of life events and interpersonal relationship were moderately correlated with IAD. Punishment, study pressure, and other negative events such as unsuccessful dating, fighting with others, scolding by parents, were weakly correlated to IAD.

There are several reasons for this phenomenon. Firstly, the social competition is increasingly fierce, which leads to certain psychological and mental pressure to people, especially adolescents and college students. They will experience more negative events, face more complicated interpersonal relationships, heavier academic tasks, and other social and family pressures which would bring more anxiety, depression, reticence, social withdrawal and other psychological and mental problems. Cyberspace as the “third space” between physical space and spiritual space (75). Its anonymity, virtuality, and concealment could be as a carrier to vent negative emotions, seek support, and escape the pressure of reality for adolescents and college students. Inappropriate long-term response has increased reliance on the internet, which has led to IAD. Kardefelt-Winther et al. (76) have proposed that IAD may represent a compensatory behavior to deal with everyday life problems and distress.

Additionally, previous empirical evidence has indicated that there were several mediating roles of negative life events and IAD (41, 45, 54). Unfortunately, the data extracted in this study did not allow the above to be used as confounding factors to look at differential correlations between life events and IAD.

In summary, negative life events and interpersonal relationship are positively associated with IAD. The Other most important factors were study pressure and punishment that should also be given more attention. In the context of the information age, the internet has become an indispensable part of our daily lives. Adolescence is a life period during which a young person often feels confused, insecure, helpless, and burdened by different expectations and demands which indicate that they may be more susceptible to IAD (77, 78). Therefore, educational institutions and government departments should put emphasis on the education of students and their individual value tendency, encouraging adolescents and college students to know right from wrong, cultivating the ability of positive response, strengthening their self-control, and resisting the basic availability of virtual network.

### Strengths and Limitations

This review has collated and analyzed published data to determine the relationship between negative life events and IAD among adolescents and college students in China. Most of the studies had large sample sizes with sufficient numbers of participants to lend power to the findings. The results might provide a new reference for the prevention and clinical intervention of IAD among adolescents and college students in China.

The limitations of our systematic review are as follows: on the one hand, there is substantial heterogeneity in the estimates of the association between life events and IAD among the studies. This heterogeneity could be due to differences in methodology between studies (including study design and research approaches) and in different professions (science students and engineering students). On the other hand, the studies of several countries focused on the impact of the internet, such as cyber harassment and bullying and were not considered valid data. Therefore, this meta-analysis represented only the studies from China. Finally, since they were observational studies, there is a risk of confounding bias even though many of the studies included techniques to control confounding.

## Conclusion

This systematic review and meta-analysis showed that there was significant positive association between negative life events and IAD, especially in interpersonal relationship among adolescents and college students in China. The findings of this review are important for educational and psychological practitioners, parents, and schools. Based on these findings intervention programs and guiding policies need to be implemented in schools and parents and family care should play a central role in these interventions. Prevention remains the cornerstone of IAD. Furthermore, it is necessary for several moderators to further the studies.

## Data Availability Statement

The original contributions presented in the study are included in the article/Supplementary Material, further inquiries can be directed to the corresponding authors.

## Author Contributions

JW and QH conceived the idea. JW drafted the manuscript. YT, WP, and HL were involved in the interpretation of the study findings. TZ, YW, and HL reviewed the manuscript and provided the comments. YW provided the language polish. All authors contributed to the article and approved the submitted version.

## Conflict of Interest

The authors declare that the research was conducted in the absence of any commercial or financial relationships that could be construed as a potential conflict of interest.

## Publisher’s Note

All claims expressed in this article are solely those of the authors and do not necessarily represent those of their affiliated organizations, or those of the publisher, the editors and the reviewers. Any product that may be evaluated in this article, or claim that may be made by its manufacturer, is not guaranteed or endorsed by the publisher.
