# Supplementary material for: The Relationship Between Negative Life Events and Internet Addiction Disorder Among Adolescents and College Students in China: A Systematic Review and Meta-Analysis
Source: Front Psychiatry. 2022 Apr 27;13:799128. doi: 10.3389/fpsyt.2022.799128 (PMC9091506; doi:10.3389/fpsyt.2022.799128)
Supplement: Supplementary file 1 [file Table_1.docx]

**Table 1** Search strategy for the PubMed database

| **Search** | **Query** |
| --- | --- |
| #7 | Search: **#3 AND #6** Sort by: **Most Recent** |
| #6 | Search: **#4 OR #5** Sort by: **Most Recent** |
| #5 | Search: **life experience [Title/Abstract]** Sort by: **Most Recent** |
| #4 | Search: **"Life Change Events"[Mesh]** Sort by: **Most Recent** |
| #3 | Search: **#1 OR #2** Sort by: **Most Recent** |
| #2 | Search: **internet addiction [Title/Abstract] OR problematic internet use [Title/Abstract] OR internet addiction disorder [Title/Abstract] OR pathological internet use [Title/Abstract] OR excessive internet use [Title/Abstract] OR compulsive internet use [Title/Abstract] OR internet dependency [Title/Abstract] OR computer addiction [Title/Abstract] OR internet use disorder [Title/Abstract]** Sort by: **Most Recent** |
| #1 | Search: **"Internet Addiction Disorder"[Mesh]** Sort by: **Most Recent** |
